# Fabry disease presenting with sudden hearing loss and otosclerosis: a case report

**DOI:** 10.1186/1752-1947-6-112

**Published:** 2012-04-16

**Authors:** Giovanni Felisati, Elisabetta Salvatici, Carlotta Pipolo, Sara Portaleone, Enrica Riva, Marcello Giovannini

**Affiliations:** 1 Department of Otorhinolaryngology, San Paolo Hospital, University of Milan, Via A. Di Rudinì 8, 20142, Milano, Italy; 2 Department of Pediatrics, San Paolo Hospital, University of Milan, Via A. Di Rudinì 8, 20142, Milano, Italy

## Abstract

**Introduction:**

Fabry disease is an X-linked lysosomal storage disorder resulting in a multiple-system disorder with a wide spectrum of physical signs and symptoms, predominantly affecting the central and peripheral nervous systems, skin, heart, kidneys, and eyes.

**Case presentation:**

We describe the case of a 26-year-old European Caucasian man who had Fabry disease and who presented with episodic sudden unilateral hearing loss and was treated with glucocorticoids, pentoxifylline, hyperbaric oxygen, and fluoride because of concomitant audiometric evidence of otosclerosis. This case demonstrates the partial and transient beneficial effect of standard treatment for sudden hearing loss not related to Fabry disease and analyzes the possible connection between typical Fabry disease inner-ear lesions and otosclerosis. Whereas hearing loss has been described in connection with Fabry disease, otosclerosis-associated hearing loss in Fabry disease has not yet been described.

**Conclusions:**

Although progressive hearing loss in patients with Fabry disease seems to be influenced by replacement therapy, few data concerning treatment of sudden hearing loss are available. The lack of literature concerning the pathogenesis of the otological involvement in Fabry disease makes it impossible to identify a connection between the latter and otosclerosis. Therefore, this report may help to reinforce the importance of a thorough evaluation of hearing in patients with Fabry disease and may be of help with therapeutic decision-making.

## Introduction

Fabry disease (FD) is an X-linked lysosomal storage disorder that is due to the deficient activity of the enzyme alpha-galactosidase A (GLA enzyme), a lysosomal hydrolase encoded by the alpha-galactosidase gene (*GLA *gene; EC 3.2.1.22) [1]. This enzyme deficiency leads to widespread deposition of neutral glycosphingolipids (mainly globotriaosylceramide and, to a lesser extent, galabiosylceramide) on blood vessel walls throughout the body, resulting in a multiple-system disorder with a wide spectrum of physical signs and symptoms that predominantly affect the central and peripheral nervous systems, skin, heart, kidneys, and eyes. Acroparesthesias and pain crises, hypohydrosis, angiokeratomas, and corneal dystrophy are among the typical initial manifestations, whereas cardiomyopathy, renal failure, and cerebrovascular events dominate in patients with FD [2]. The otorhinolaryngological area is not spared by this disease, and the majority of patients develop progressive and accelerated sensorineural hearing loss (SNHL) during adulthood [3]. Also, a high incidence of vestibular disorders with dizziness and chronic instability is observed in these patients [4].

Otosclerosis (incidence of 0.3% to 0.4% in Caucasians) [5] is a bone remodeling disorder of the human otic capsule; however, the etiopathogenesis remains unclear. Genetic predisposition, disturbed bone metabolism, persistent measles virus infection, autoimmunity, and hormonal and environmental factors may play contributing roles in the pathogenesis of otosclerosis. Clinical signs are progressive conductive hearing loss or SNHL (or both) as a consequence of stapes footplate fixation and cochlear bone resorption with endosteal involvement. Instrumental examinations (audiometry, impedensitometry, and computed tomography) aid in identifying affected patients, but a definitive diagnosis can be obtained only through surgical inspection of the middle ear, confirming fixation of the stapes.

We describe the case of a 26-year-old Caucasian man who had FD and who presented after two episodes of unilateral sudden hearing loss (SHL) and who was treated with glucocorticoids, pentoxifylline, and hyperbaric oxygen without complete recovery of right ear function. The case was associated with the audiometric characteristics of otosclerosis.

## Case presentation

A 26-year-old Caucasian man tested positive for FD when he was nine years old. At presentation, his main manifestations were fatigue, hypohydrosis, and painful acroparesthesias correlated with fever and physical effort.. The dosage of GLA activity in lymphocytes was 2.4 nmol/hour per mg, which was well below normal values (24 to 56 nmol/hour per mg protein), and a molecular genetic analysis confirmed the diagnosis (R100K mutation in exon 2). Our patient was in good health until 18 years of age, when he had three episodes of severe nephrotic syndrome and acute renal failure requiring hospitalization and corticotherapy. Enzyme replacement therapy (ERT) with agalsidase-β (Fabrazyme^®^; Genzyme Corporation, Cambridge, MA, USA), at a dosage of 1 mg/kg intravenously every 2 weeks, was started in 2001 after approval of the drug in Italy. Since then, his renal function has been stable. Before the start of ERT, the brain (evaluated by magnetic resonance imaging), fundus oculi, cardiac function and morphology, hearing, and kidney function were evaluated, and all were normal.

Almost four years ago, our patient complained of dizziness lasting 30 to 40 minutes with spontaneous regression associated with left-sided SHL (as defined by Hughes and colleagues [6]) and tinnitus. The results of a neurological examination were normal, as were those of an audiogram performed a few weeks before the onset of symptoms. Also, our patient had no history of SHL, otosclerosis, or any other auditory disease in his family.

**Figure 1:**
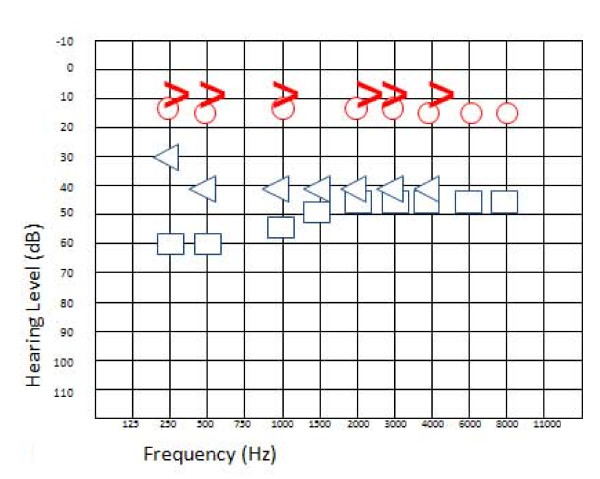
**Left sensorineural hearing loss associated with a mild conductive component on low frequencies after the onset of symptoms**. Blue cross, left ear conduction; blue open arrow, left ear bone conduction; blue square, left ear conduction with narrow band note masking on the opposite ear; blue triangle, left ear bone conduction with narrow band note masking on the opposite ear; red circle, right ear air conduction; red open arrow, right ear bone conduction.

We then performed an audiometric test, which showed a left SNHL associated with a mild conductive component at low frequencies (Figure 1) and a normal (type A) tympanogram without stapedial reflexes (ipsilateral and contralateral) with the probe in the left ear. The result of pure-tone audiometry was normal in the right ear. Further examination showed vestibular suffering of ischemic etiology in all likelihood and a left-beating nystagmus revealed only by the head-shaking test and increased by lateral decubitus.

An otological investigation revealed normal tympanic membranes. The brainstem auditory evoked response was consistent with the auditory impairment, showing increased latencies for the central pathways of the left ear, and an augmented I-V interval was on the left side.

Our patient presented no other associated symptoms.

**Figure 2:**
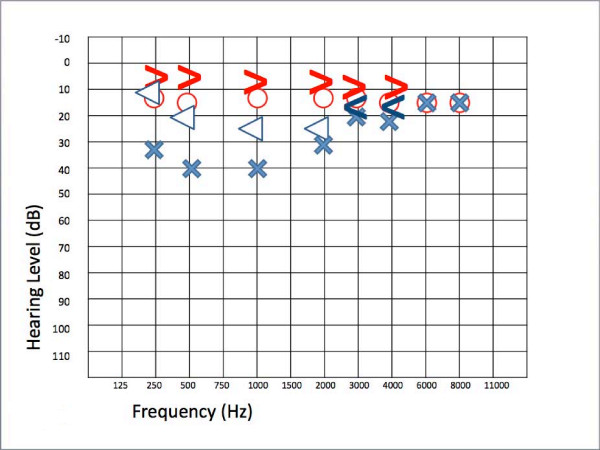
**Almost complete normalization of bone conduction with only a mild deficit (threshold of 25dBnHL at 1 to 2 kHz)**. An air-bone gap on low frequencies remained after therapy. Blue cross, left ear conduction; blue open arrow, left ear bone conduction; blue square, left ear conduction with narrow band note masking on the opposite ear; blue triangle, left ear bone conduction with narrow band note masking on the opposite ear; red circle, right ear air conduction; red open arrow, right ear bone conduction.

Oral corticosteroid therapy with prednisone (50 mg/day for two days decreased to 25 mg every two days for eight days) along with hyperbaric oxygen therapy was administered for eight days; our patient underwent eight sessions of 80 minutes each at 1.5 bars of pressure with 100% oxygen. Audiometry was performed after eight days and showed mild recovery of both the bone and air conduction. However, our patient continued to complain of left-ear hearing loss associated with intense tinnitus. This prompted us to continue therapy by performing a second cycle of hyperbaric oxygen treatment with the addition of oral pentoxifylline (400 mg twice per day for 15 days) as an anti-aggregant. Our patient reported subjective improvement of the tinnitus after the fifth session. An ear, nose, and throat evaluation after the completion of therapy showed an almost complete normalization of bone conduction and only a mild deficit, whereas a conductive gap on low frequencies remained (Figure 2), calling into question the purely vascular origin of the hearing loss. The brain and cerebellopontine angle were examined by magnetic resonance imaging to detect the persistence of the left interventricular trigonal lesion, present for six years, and a new malacic lesion at the left thalamic site.

**Figure 3:**
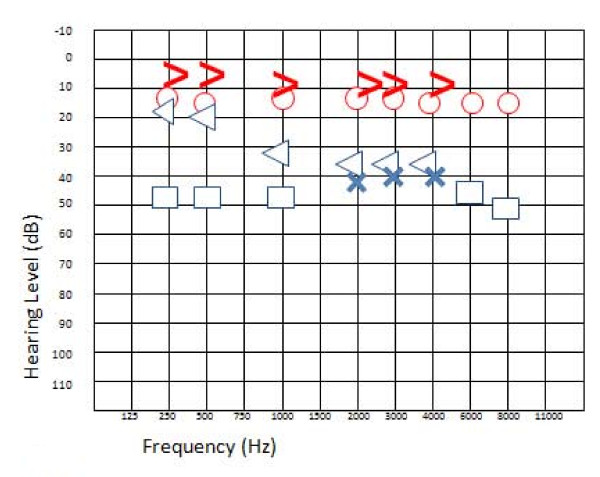
**Audiogram of the second crisis**. Hearing function is decreased for frequencies of 2 to 8 kHz. The air-bone gap remains unchanged. Blue cross, left ear conduction; blue open arrow, left ear bone conduction; blue square, left ear conduction with narrow band note masking on the opposite ear; blue triangle, left ear bone conduction with narrow band note masking on the opposite ear; red circle, right ear air conduction; red open arrow, right ear bone conduction.

Our patient experienced a new SHL crisis and tinnitus about eight months later. An audiogram showed a decrease in hearing function for frequencies of 2 to 8 kHz, but the conductive gap was unchanged (Figure 3). Our patient presented a type A tympanogram with no evocable stapedial reflexes on the left side.

As the audiometric pattern resembled that of otosclerosis, we performed a fine-slice computed tomography scan to study the oval window, but no alterations were found. Our patient underwent the same multi-drug (prednisone and pentoxifylline) and hyperoxygen therapy with the addition of sodium fluoride (30 mg/day orally) for two months to slow down the evolution of the suspected otosclerosis.

**Figure 4:**
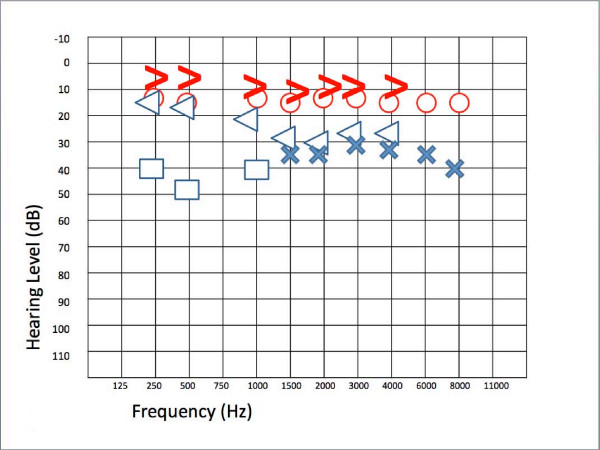
**Partial recovery of high-frequency hearing three months after the onset of the second crisis**. Blue cross, left ear conduction; blue open arrow, left ear bone conduction; blue square, left ear conduction with narrow band note masking on the opposite ear; blue triangle, left ear bone conduction with narrow band note masking on the opposite ear; red circle, right ear air conduction; red open arrow, right ear bone conduction.

Repeated audiograms performed after five days and one month showed no significant recovery, whereas the audiogram performed three months after the onset of the hearing loss showed partial recovery of high-frequency hearing (Figure 4). However, the conductive component did not change and the suspicion of otosclerosis remained.

Given the increased risk of stroke and microvascular disease, our patient began permanent therapy with acetyl salicylic acid at a dosage of 300 mg/day. Brainstem auditory evoked potentials performed during and three months after the second episode showed mild suffering of the central auditory pathway on the left. Minor SNHL with associated conductive hearing loss remained at the two-year follow-up.

## Discussion

This case of FD presents the unusual association of two different complex audiological diseases: otosclerosis combined with SHL. Otosclerosis is a well-defined entity and presents two different treatment options. Medical therapy for otosclerosis can slow down the progression of hearing loss, but the only possible cure to restore hearing is a surgical stapedotomy [7]. As stated before, the incidence of otosclerosis in patients with FD has never been analyzed.

Several studies have indicated that progressive hearing loss in patients with FD comprises 50% of the morbidity, but only one study showed an SHL incidence of 11.8% in men with FD [8]. Conti and Sergi [9] reported hearing loss and vestibular function disorders in a cohort of 14 patients and a high incidence of sudden onset or progression (or both) of symptoms. Hajioff and colleagues [10] studied 15 patients and also reported some cases of conductive hearing loss.

More recently, two large studies on hearing loss in patients with FD and on the effect of ERT have been published. Hegemann and colleagues [11] analyzed the audiograms of 86 patients and found that hearing in patients with FD is significantly impaired with respect to the age-matched general population and that this leads to clinically relevant hearing impairment in 16% of cases.

Hearing loss in patients with FD typically involves all frequencies and appears to be more rapidly progressing than the hearing loss associated with normal aging. In men, residual enzyme activity of greater than 1.5% appears to have a protective function, and ERT can stabilize and possibly improve progressive hearing loss but has no proven effect on preventing SHL [8]. Ries and colleagues [8] described a positive association between hearing loss and the degree of peripheral neuropathy and cerebrovascular and renal damage, suggesting a combined neuropathic and vascular etiology of the disease. However, only a few data concerning treatment of SHL in patients with FD are available.

ERT was successful for improving and stabilizing renal function in our patient. Consistent with what is stated in the literature, areas not responsive to ERT were the central nervous system and the auditory apparatus.

## Conclusions

As this case report demonstrates, there is only a partial and transient beneficial effect of ERT and hyperbaric oxygen treatment combined with corticotherapy and pentoxifylline for hearing loss in patients with FD. As there is no international consensus on treatment of SHL [12] (particularly in patients with FD), the administered therapy is the standard therapy for SHL, and the results obtained in our case were at their best for the first treatment course, whereas partial regression of the symptoms occurred on the second occasion. Although SHL in patients with FD is rarely described, the symptoms are consistent with the vascular alterations caused by the disease in other areas. The incidence of SHL may be underestimated.

The mechanism of progressive SHL in patients with FD remains unclear. It has been suggested that globotriaosylceramide accumulation in vascular endothelium, decreased numbers of spiral ganglion cells [13], long-term obstruction of cranial vessels by dolicho-ectasic arteries because of the local deposition of glycosphingolipids into the wall of arteries, and atrophy of the stria vascularis and spiral ligament might concur with the genesis of otoneurological symptoms [14].

Also, the etiology of SHL is difficult to demonstrate even in otherwise healthy patients because the involved anatomical structures may be evaluated only partially *in vivo*. Patients with recurrent SHL usually present with punctiform cerebral white matter lesions representing microvascular brain damage, as is the case in patients with FD.

To the best of our knowledge, no previous description of otosclerosis in association with FD is available. We could not find an obvious link between otosclerosis and FD, as hypervascularization has been described for otosclerosis in contrast to the microvascular damage seen in FD. However, the involved structures are the same and the spiral ligament has been described in both otosclerosis and FD atrophy of the stria vascularis [14,15].

Further structured studies are necessary to identify the underlying mechanism of inner-ear involvement in patients with FD. Also, a careful follow-up is essential to evaluate the degree of cochlear involvement, understand the fundamental pathophysiological mechanisms, and prevent SHL and the irreversible progression of hearing damage in patients who have already undergone ERT.

## Abbreviations

ERT: enzyme replacement therapy; FD: Fabry disease; GLA: alpha-galactosidase A; SHL: sudden hearing loss; SNHL: sensorineural hearing loss.

## Consent

Written informed consent was obtained from the patient for publication of this case report and accompanying images. A copy of the written consent is available for review by the Editor-in-Chief of the journal.

## Competing interests

The authors declare that they have no competing interests.

## Authors' contributions

MG established the pharmacological and therapeutic approaches to the metabolic disease and reviewed the manuscript. ER assessed the overall condition of the patient and contributed the pediatric part of the manuscript. ES was involved in the follow-up of the patient and critically revised the manuscript. GF was involved in the therapeutic approach of the sudden hearing loss and provided a major review of the ear, nose, and throat part of the manuscript. SP performed the audiograms and the otological follow-up and contributed to the writing of the manuscript. CP performed the literature search and wrote the manuscript. All authors read and approved the final manuscript.
